# Nicorandil alleviates apoptosis in diabetic cardiomyopathy through PI3K/Akt pathway

**DOI:** 10.1111/jcmm.14413

**Published:** 2019-05-26

**Authors:** Xuyang Wang, Jinyu Pan, Dian Liu, Mingjun Zhang, Xiaowei Li, Jingjing Tian, Ming Liu, Tao Jin, Fengshuang An

**Affiliations:** ^1^ The Key Laboratory of Cardiovascular Remodeling and Function Research, Chinese Ministry of Education, Chinese National Health Commission and Chinese Academy of Medical Sciences, The State and Shandong Province Joint Key Laboratory of Translational Cardiovascular Medicine, Department of Cardiology Qilu Hospital of Shandong University Jinan China; ^2^ Department of Cardiology Shandong Provincial Qianfoshan Hospital of Shandong University Jinan China

**Keywords:** apoptosis, diabetic cardiomyopathy, nicorandil, PI3K/Akt signal

## Abstract

Nicorandil exerts myocardial protection through its antihypoxia and antioxidant effects. Here, we investigated whether it plays an anti‐apoptotic role in diabetic cardiomyopathy. Sprague‐Dawley rats were fed with high‐fat diet; then single intraperitoneal injection of streptozotocin was performed. Rats with fasting blood glucose (FBG) higher than 11.1 mmol/L were selected as models. Eight weeks after the models were built, rats were treated with nicorandil (7.5 mg/kg day and 15 mg/kg day respectively) for 4 weeks. H9c2 cardiomyocytes were treated with nicorandil and then stimulated with high glucose (33.3 mmol/L). TUNEL assay and level of bcl‐2, bax and caspase‐3 were measured. 5‐HD was used to inhibit nicorandil. Also, PI3K inhibitor (Miltefosine) and mTOR inhibitor (rapamycin) were used to inhibit PI3K/Akt pathway. The results revealed that nicorandil (both 7.5 mg/kg day and 15mg/kg day) treatment can increase the level of NO in the serum and eNOS in the heart of diabetic rats compared with the untreated diabetic group. Nicorandil can also improve relieve cardiac dysfunction and reduce the level of apoptosis. In vitro experiments, nicorandil (100 µmol) can attenuate the level of apoptosis stimulated by high glucose significantly in H9C2 cardiomyocyte compared with the untreated group. The effect of nicorandil on apoptosis was blocked by 5‐HD, and it was accompanied with inhibition of the phosphorylation of PI3K, Akt, eNOS, and mTOR. After inhibition of PI3K/Akt pathway, the protective effect of nicorandil is restrained. These results verified that as a NO donor, nicorandil can also inhibit apoptosis in diabetic cardiomyopathy which is mediated by PI3K/Akt pathway.

## 1 INTRODUCTION

The incidence of diabetes is increasing through the years. Its related complications have threatened the survival rate and life quality of diabetic patients.^1^ Diabetes is a risk factor for cardiovascular disease and diabetic cardiomyopathy (DCM) is a major complication in diabetic patients.^2^, ^3^ Compromised left ventricular contraction or diastolic dysfunction and ventricular hypertrophy are the main features of DCM, independent of hypertension, coronary syndrome and other diseases.^4^ Increase of cardiomyocytic apoptosis and deposition of extracellular matrix protein, which are caused by hyperglycaemia and metabolic disturbance are the main pathological changes of heart in diabetes, leading to the development of DCM.^5^, ^6^

Myocardial apoptosis plays a vital role in pathogenesis of cardiovascular diseases in the diabetes.^7^ It is known that PI3K/Akt pathway can inhibit apoptosis.^8^ When PI3K/Akt pathway is blocked in the heart of the diabetic rats, the protein expression of eNOS and mTOR which can regulate the level of apoptosis is reduced significantly.^9^, ^10^ Thus, exploring the underlying mechanism of how PI3K/Akt pathway is mediated and finding effective drugs which can target on cardiomyocyte apoptosis can alleviate the process of DCM.

Nicorandil is an antianginal agent that causes vasodilatation by dual action: one is releasing nitric oxide (NO) and the other is binding to ATP‐dependent K channel and opening it.^11^, ^12^ Recent studies show that nicorandil can reduce the level of reactive oxygen species(ROS) in endothelial cells and regulate the PI3K/Akt pathway.^13^, ^14^, ^15^ However, whether it can alleviate cardiomyocyte apoptosis in diabetic cardiomyopathy has not been reported yet. In the present study, we investigated the myocardial protective effect of nicorandil on DCM in streptozotocin (STZ)‐induced diabetic rats by adjusting the level of cardiac function and remodelling. The possible mechanism underlying the protective effect of nicorandil was also investigated in apoptotic levels of the rats' heart. The potential molecular mechanism was investigated in H9C2 cardiomyocyte.

## 2 METHODS AND MATERIAL

### 2.1 Animal

Sixty Sprague‐Dawley rats （100‐120 g） were randomly allocated into four groups （n = 15). All rats were kept with a light‐dark cycles at 23°C. The control group were fed with the basal diet and the other three groups were fed with high‐fat diet (HF diet; 16% fat and 0.30% cholesterol). Four weeks after the HF diet, we performed intraperitoneal insulin tolerance test (IPITT) and intraperitoneal glucose tolerance test (IPGTT) to indentify insulin‐resistant rats. Single intraperitoneal injection of streptozotocin (STZ;40 mg/kg, solarbio, China) to rats with insulin resistance was performed to induce the diabetic models. Then we measured the fasting blood glucose (FBG) 7 days after the injection. Only rats with FBG ≥11.1 mmol/L were considered as a successful diabetic model.^16^, ^17^ After 8 weeks of high blood glucose, the rats were given nicorandil through drinking water at the concentration of 7.5 mg/kg day and 15 mg/kg day respectively. To constantly administer the amount of nicorandil, the concentration of nicorandil in the drinking water was adjusted every 4 days along with as per the water intake volume. We performed IPITT and IPGTT and killed all rats 4 weeks later after nicorandil treatment. All experimental protocols were approved by the law of Shandong University Animal Care Committee.

**Table 1:** Basic information of rats

	Control	DM	DM + N7.5	DM + N15
FBG (mmol/L)	5.06 ± 0.45	21.10 ± 0.81*	20.20 ± 0.38*	22.13 ± 0.62*
TC (mmol/L)	1.63 ± 0.08	2.89 ± 0.05*	2.81 ± 0.09*	2.92 ± 0.04*
TG (mmol/L)	0.58 ± 0.03	2.73 ± 0.06*	2.76 ± 0.08*	2.71 ± 0.04*
INS (mmol/L)	14.05 ± 0.32	16.17 ± 0.43*	16.34 ± 0.38*	16.09 ± 0.29*
ISI (mmol/L)	−3.88 ± 0.04	−5.21 ± 0.05*	−5.27 ± 0.02*	−5.36 ± 0.08*
SBP (mm Hg)	117.6 ± 4.4	121.9 ± 3.8	119.3 ± 4.5	119.6 ± 5.3
DBP (mm Hg)	91.5 ± 4.3	92.4 ± 2.8	93.4 ± 5.9	91.6 ± 3.5
MBP (mm Hg)	99.4 ± 2.5	102.9 ± 4.8	101.7 ± 3.4	103.8 ± 2.4

Abbreviations: DBP, dialated blood pressure; FBG, Fasting blood glucose; INS, insulin; ISI, insulin sensitive index; MBP, median blood pressure; SBP, systolic blood pressure; TC, total cholesterol; TG, total triglyceride.

* *P* < 0.05 compared with the control group.

### 2.2 Cardiac function

Cardiac function of rats was measured by the Vevo 770 imaging system with the RMB710 transducer (VisualSonics, Toronto, Canada). The echocardiography parameters involved the left ventricular end‐diastolic dimension (LVEDd), left ventricular ejection fraction(LVEF), peak E to peak A ratio(E/A), early (e’) to late (a’) diastolic velocity ratio(e/a), peak E to early (e’) ratio(E/e’) and the fractional shortening(FS).

### 2.3 Histology staining

Hearts were fixed with the 4% paraformaldehyde and embedded with paraffin, which were sliced to 4 µm for haematoxylin and eosin (HE) staining. We performed masson's trichrome and sinus red to measure the level of fibrosis. We used the antibody of collagen III (novusbio, NB600‐594SS), collagen I (novusbio, NBP1‐30054) for immunohistochemistry. Briefly, primary antibody of collagen I and collagen III were used to incubate the sections overnight at 4°C overnight and then sections were washed with phosphate buffered saline (PBS) and incubated in secondary antibody for 30 min in 37°C.^18^

### 2.4 Cell treatment

H9c2 cardiomyocytes were plated in the six‐well plated(Corning) in Dulbecco's modified Eagle's medium (DMEM, glucose 5.5 mM) supplemented with 10% foetal bovin serum(FBS) and 1% penicillin‐streptomycin at 37°C in the 5% CO2 for more than 12 hr. When the cells reached 60% confluence, the minimal essential medium was substituted with medium of glucose (33.3 mM）, the osmotic pressure was balanced by mannitol and stimulated for 24 hr. Meanwhile, nicorandil with different concentrations (N1: 10 µmol; N2: 50 µmol; N3:100 µmol) was added in the HG‐treated group. 5‐hydroxydecanoate (5‐HD, Sigma‐Aldrich, USA) was used to inhibit nicorandil at the time of nicorandil treatment in HG stimulation for 24 hr. Miltefosine (MTF, MCE, USA) and rapamycin (Rapa, MCE, USA) were used to inhibit PI3K/Akt/mTOR pathway at the time of nicorandil treatment in HG stimulation for 24 hr.

### 2.5 TUNEL staining

TUNEL assay was used at the cell apoptosis assay.^19^ For DNA fragmentation, TUNEL assays kit (Millipore, USA) was used to treat the H9c2 cardiomyoblasts or tissue sections according to manufacturer's instructions. Briefly, cells were fixed with 4% paraformaldehyde for 10 min at room temperature and then washed with PBS twice. Paraffin tissue sections were treated with 20 µg/ml proteinase K for 5 min. Then the samples were treated with the 3% H_2_O_2_ for 15 min and incubated with 10 µl TdT enzyme reaction buffer for 1 hr at 37°C. After incubation, digoxigenin antibody buffer was added before DAB incubation. PBS was used to wash residual buffer one last time.

### 2.6 Western blot

Protein from rat heart or cells was separated through 10% sodium dodecyl sulphate polyacrylamide gel electrophoresis (SDS‐PAGE) and transferred to PVDF membrane (Millipore, USA). Five percent non‐fat milk was used to block the membrane for 1 hr at room temperature and then the protein on the membrane was incubated overnight with primary antibody at 4°C. Secondary antibodies were used for 1 hr at room temperature and enhanced chemiluminescence (Millipore, USA) was used for exposure via Amersham Imager 600 (General Electric Company, USA).We used the antibody against bcl‐2(abcam, ab180665), bax(novusbio, NBP1‐78977SS), cleaved caspase‐3(abcam, ab216995), caspase‐3(abcam, ab216995), collagenIII (novusbio, NB600‐594SS), collagenI (novusbio, NBP1‐30054), MMP2 (Proteintech, 10373‐2‐AP), MMP9 (Proteintech, 10387‐2‐AP), p‐PI3K (CST, 4228), PI3K (CST, 4292), p‐Akt (abcam, ab38449), Akt (abcam, ab179463), p‐mTOR (abcam, ab109268), mTOR (abcam, ab2732), p‐eNOS (abcam, ab230158), eNOS (CST,32027) and β‐actin (abcam, ab8227) for detection.

### 2.7 Statistical analysis

All analyses were carried out in Prism 6.0 (Graphpad) and SPSS 20.0. One‐way ANOVA was used to compare the difference among groups and unpaired t‐test was used for the difference between two groups. Each experiment was repeated at least three times and data were shown as means ± standard deviation (SD). Two‐tailed *P* < 0.05 was treated as statistically significant.

## 3 RESULTS

### 3.1 Basic characteristic of type 2 diabetic rats

**Figure 1:**
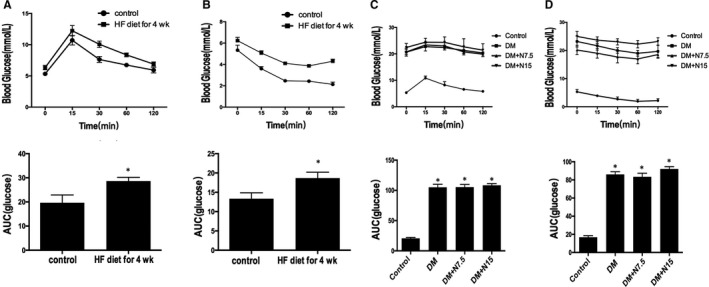
Characteristic of type 2 diabetic rats. IPGTT(A) and IPITT(B) were performed after 4 weeks of high‐fat diet. AUC were calculated in control and HF group. After nicorandil treatment for 4 weeks, IPGTT(C) and IPITT(D) were performed in four groups. AUC were calculated in 4 groups. *P < 0.05 compared with the control group

We performed intraperitoneal insulin tolerance test (IPITT) and intraperitoneal glucose tolerance test (IPGTT) to rats after high‐fat diet for 4 weeks. Insulin resistance occurred in the high‐fat diet (HF) group，the mean area under the receiver operating characteristic curve (AUC) increased significantly in HF group compared with the control group (Figure 1). Then insulin‐tolerant rats were injected intraperitoneally with STZ. IPITT and IPGTT experiments were performed among groups after treatment of nicorandil for 4 weeks. There was no significant difference in blood glucose between the nicorandil group and untreated DM group. The blood pressure of the untreated DM group appeared to be higher than that of the nicorandil group, but this difference did not reach statistical significance (Table 1).

**Figure 2:**
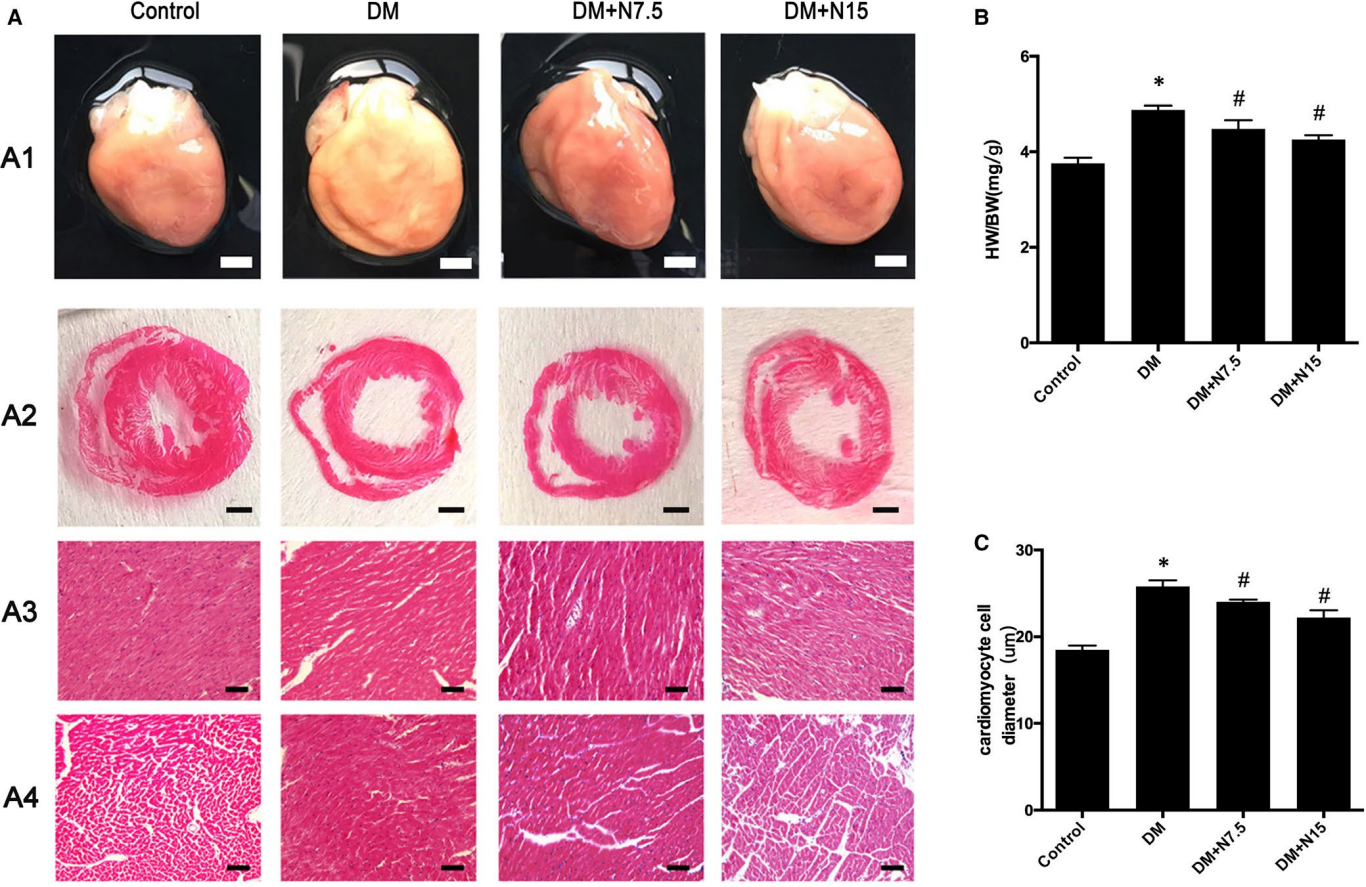
Nicorandil alleviates myocardial remodelling in diabetic rats. A: A1: gross morphology; A2: HE staining of cross shaft of musculi papillares in heart A3: HE staining longitudinal section; A4: HE staining of cross section; B: heart weight/bodyweight; C: cardiomyocyte cell diameter. DM: Diabetic mellitus. N7.5: nicorandil, 7.5 mg/kg·day; N15: nicorandil, 15 mg/kg·day. *P < 0.05 compared with control; ^#^ *P* < 0.05 compared with DM; Data are means ± SD

### 3.2 Nicorandil alleviated left ventricular hypertrophy and myocardial structural changes in rats with diabetes

Compared with the control group, the heart of rats with diabetes presented the characteristic of pathological hypertrophy, the ratio of heart weight to bodyweight increased, and the diameter of cardiomyocytes increased significantly. Compared with the untreated group, both treatments by nicorandil with the concentration of 7.5 mg/kg day and 15 mg/kg day can reduce the ratio of heart weight to bodyweight and alleviate ventricular hypertrophy (Figure 2A and 2). Also, the diameter of cardiomyocytes in rats decreased significantly after nicorandil treatment （Figure 2C).

### 3.3 Protective effects of nicorandil on heart contractile and diastolic dysfunction

**Figure 3:**
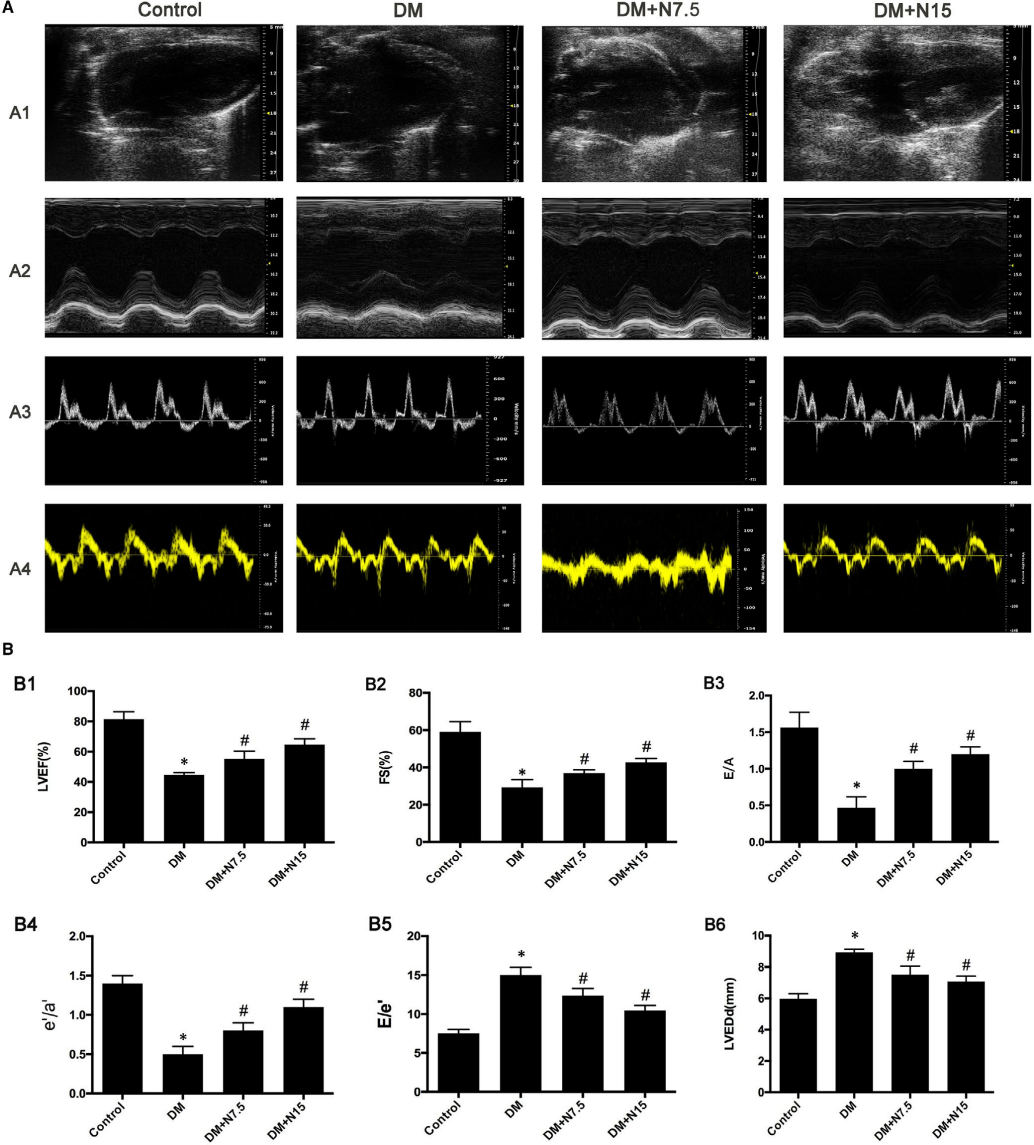
Nicorandil alleviates cardiac dysfunction in diabetic rats. A1: representive 2D echocardiograms. A2: representative M‐mode echocardiograms. A3: representative pulse‐wave Doppler echocardiograms of mitral inflow. A4: representative tissue Doppler echocardiograms. B1: Left ventricle ejection fraction (LVEF). B2: Fractional shortening (FS). B3: Early to late mitral flow (E/A). B4: Ratio of diastolic mitral annulus velocities (e’/a’). B5: E/e’. B6: Left ventricle end‐diastolic dimension (LVEDd). DM: Diabetic mellitus. N7.5: nicorandil, 7.5 mg/kg·day; N15: nicorandil, 15 mg/kg·day. *P < 0.05 compared with control; ^#^ *P* < 0.05 compared with DM; Data are means ± SD

Cardiac function in DM rats was examined by ultrasound. Compared with the control group, the LVEF, LVEDD, F/S, e/a, E/A and E/e indicators of DM rats were abnormal, showing left ventricular systolic and diastolic dysfunction. Although, cardiac dysfunction was improved in rats with diabetes after nicorandil treatment compared with untreated group (Figure 3). Both the concentration of 7.5 mg/kg day and 15 mg/kg day have this protective effect.

### 3.4 Nicorandil relieved myocardial fibrosis and apoptosis in heart of rats with diabetes

**Figure 4:**
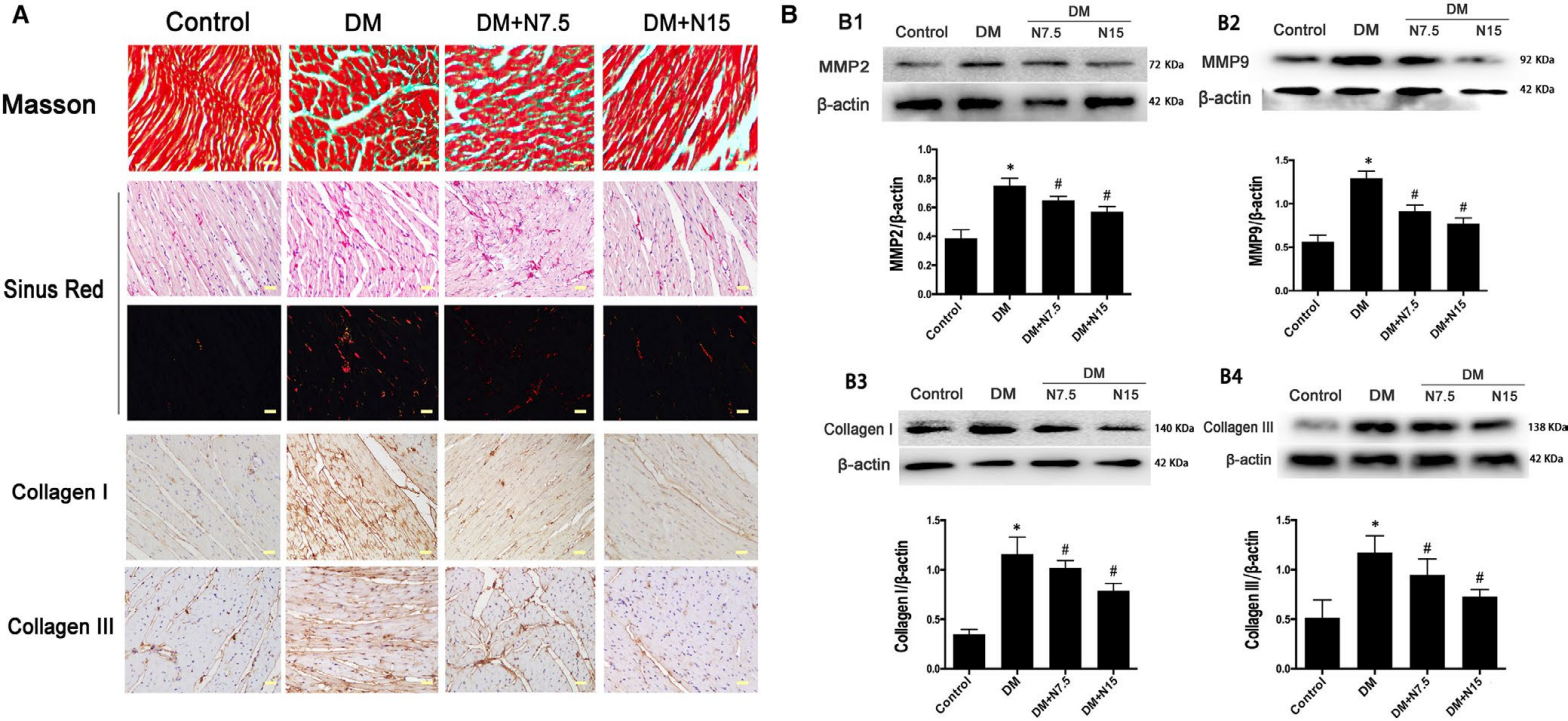
Nicorandil alleviate cardiac fibrosis in type 2 diabetic rat. A: Masson's trichrome staining and Picorosirius Red staining of myocardium. Immunohistochemical staining of Collagen I and Collagen III; B: Western blot analysis of MMP2, MMP9, Collagen I and Collagen III. DM: Diabetic mellitus, N7.5: nicorandil, 7.5 mg/kg·day; N15: nicorandil, 15 mg/kg·day. *P < 0.05 compared with control; ^#^ *P* < 0.05 compared with DM; ^#^ *P* < 0.05 compared with HG + N, Data are means ± SD

Compared with the control group, masson and sirius red staining showed an increased level of extracellular matrix in the interstitial region of the DM group. The area of fibrosis between cardiomyocytes increased, and the expression of collagen I, collagen III, MMP2 and MMP9 increased significantly. Myocardial fibrosis levels are reduced after treatment with nicorandil (Figure 4).

**Figure 5:**
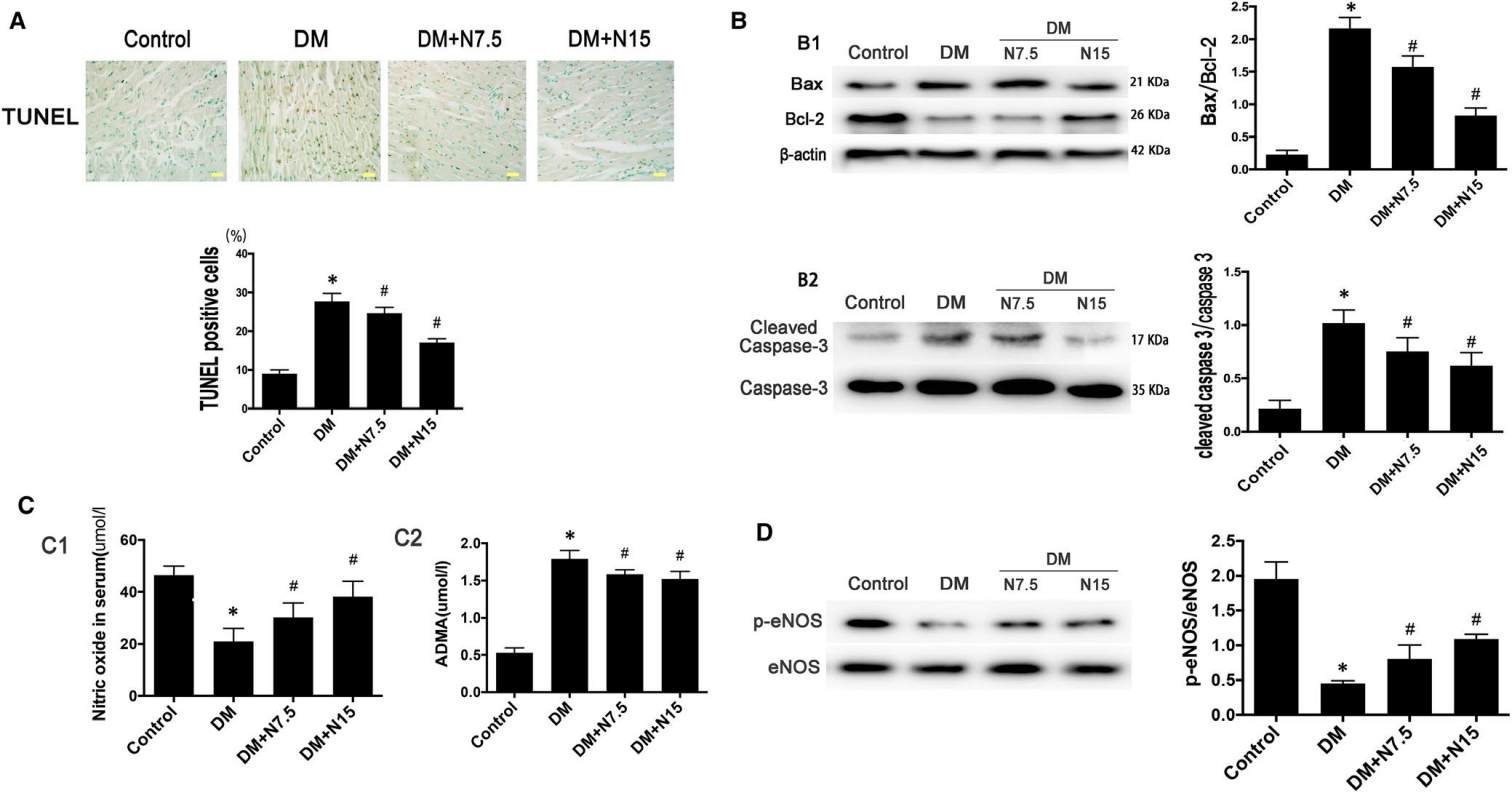
Nicorandil alleviates cardiac apoptosis in type 2 diabetic rat. A: TUNEL staining and TUNEL‐positive cells rate. B: Western blot analysis of Bax/Bcl‐2 and cleaved caspase‐3. C: Level of nitric oxide and ADMA in serum. D: Western blot analysis of p‐eNOS. DM: Diabetic mellitus, N7.5: nicorandil, 7.5 mg/kg·day; N15: nicorandil, 15 mg/kg·day. *P < 0.05 compared with control; ^#^ *P* < 0.05 compared with DM; ^#^ *P* < 0.05 compared with HG + N, Data are means ± SD

Compared with the control group, TUNEL staining showed an increase in the level of cardiomyocyte apoptosis in the DM group, whereas the rate of TUNEL‐positive cells were decreased after nicorandil treatment (Figure 5A). And the ratio of Bax/Bcl‐2 and cleaved‐caspase‐3/caspase‐3 in DM group increased significantly compared with the control group. The expression of bax and cleaved caspase‐3 decreased and the bcl‐2 increased in the heart of nicorandil‐treated group compared with the diabetic rats with no treatment (Figure 5B). Additionally, we measured the level of NO and ADMA in serum; the DM group showed a decrease of NO which was accompanied with the increase of ADMA compared with the control group, whereas nicorandil can increase the NO significantly and also reduce the ADMA to a certain extent (Figure 5C). Also, the p‐eNOS/eNOS ratio in heart increased significantly after the nicorandil treatment compared with the untreated DM group (Figure 5D).

### 3.5 Nicorandil can alleviate apoptosis effectively in H9c2 cardiomyocyte treated with high glucose

**Figure 6:**
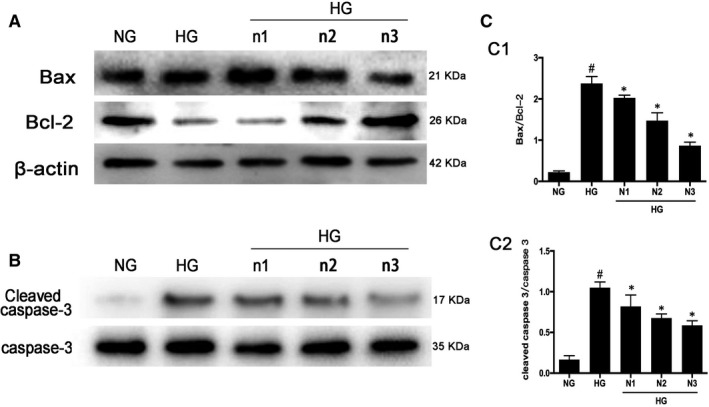
Apoptosis level reduced after nicorandil treatment in high glucose‐induced H9c2 cardiomyocyte. A: Western blot analysis of bax and bcl‐2 in high glucose‐induced H9c2 cardiomyocyte after nicorandil treatment with different concentrations for 24 h. B: Western bolt analysis of cleaved caspase‐3 in high glucose‐induced H9c2 cardiomyocyte after nicorandil treatment. HG (33.3 mmol/L), NG (5.5 mmol/L), n1: nicorandil (10 µmol); n2: nicorandil (50 µmol); n3: nicorandil (100 µmol). ^#^ *P* < 0.05 compared with NG; *P < 0.05 compared with HG, Data are means ± SD

H9C2 cardiomyocyte is cultured in DMEM with low glucose (1 g/L) and then treated with high glucose (HG, 33 mmol/L). The level of apoptosis increased significantly after HG stimulation which was manifested by the increased ratio of Bax/Bcl‐2 and cleaved‐caspase‐3/caspase‐3. Nicorandil (n1:10 µmol; n2:50 µmol; n3:100 µmol) treatment was performed before the high‐glucose treatment. After nicorandil treatment in cells, the ratio of Bax/Bcl‐2 and cleaved‐caspase‐3/caspase‐3 was decreased significantly (Figure 6).

### 3.6 Nicorandil relieves apoptosis in H9c2 cardiomyocyte treated with high glucose and 5‐HD can block this function

5‐HD acts as a competitive antagonist of nicorandil, which blocks the binding of nicorandil to the receptor. After the addition of 5‐HD (500 µmol) to H9c2 cardiomyocyte for 1 hr, the nicorandil (100 µmol) was added. Nicorandil treatment can alleviate the level of apoptosis in H9c2 cells treated with HG and increase the phosphorylation of PI3K, AKT, eNOS and mTOR. The level of apoptosis increases after 5‐HD treatment and the phosphorylation of PI3K, AKT, eNOS and mTOR reduced compared with the nicorandil‐treated group (Fi7).

**Figure 7:**
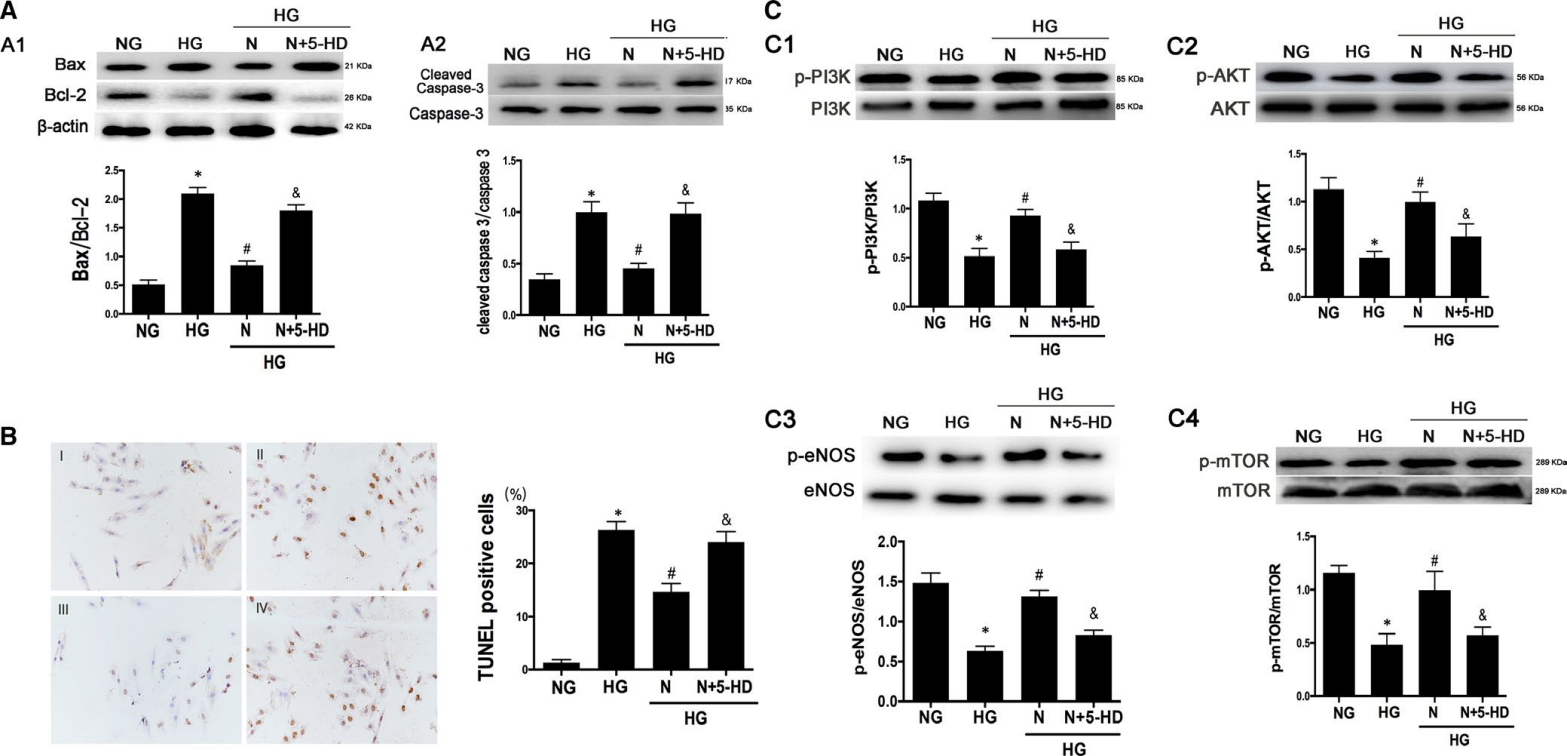
Nicorandil protects H9C2 cells from apoptosis through PI3K/AKT pathway. A: Western blot analysis of Bax/Bcl‐2 and cleaved caspase‐3 in high glucose‐induced H9c2 cardiomyocyte after nicorandil treatment or both nicorandil treatment and 5‐HD which is a inhibitor of nicorandil. B: TUNEL assay of apoptosis rate of high glucose‐induced H9c2 cardiomyocyte after nicorandil treatment or both nicorandil treatment and nicorandil inhibitor（5‐HD, 500 µmol) (scale bar: 20 µm). I:NG, II:HG, III:HG + N, IV:HG + N+5‐HD; C: Western blot analysis of phosphorylation level of PI3K, AKT, eNOS and mTOR in high glucose‐induced H9c2 cardiomyocyte after nicorandil treatment or both nicorandil treatment and nicorandil inhibitor（5‐HD). N: Nicorandil (100 µmol); NG: normal glucose (5.5 mmol/L); HG: high glucose (25 mmol/L). *P < 0.05 compared with NG; ^#^ *P* < 0.05 compared with HG; & *P* < 0.05 compared with HG + N, Data are means ± SD

### 3.7 Nicorandil relieves apoptosis through PI3K/AKT pathway

**Figure 8:**
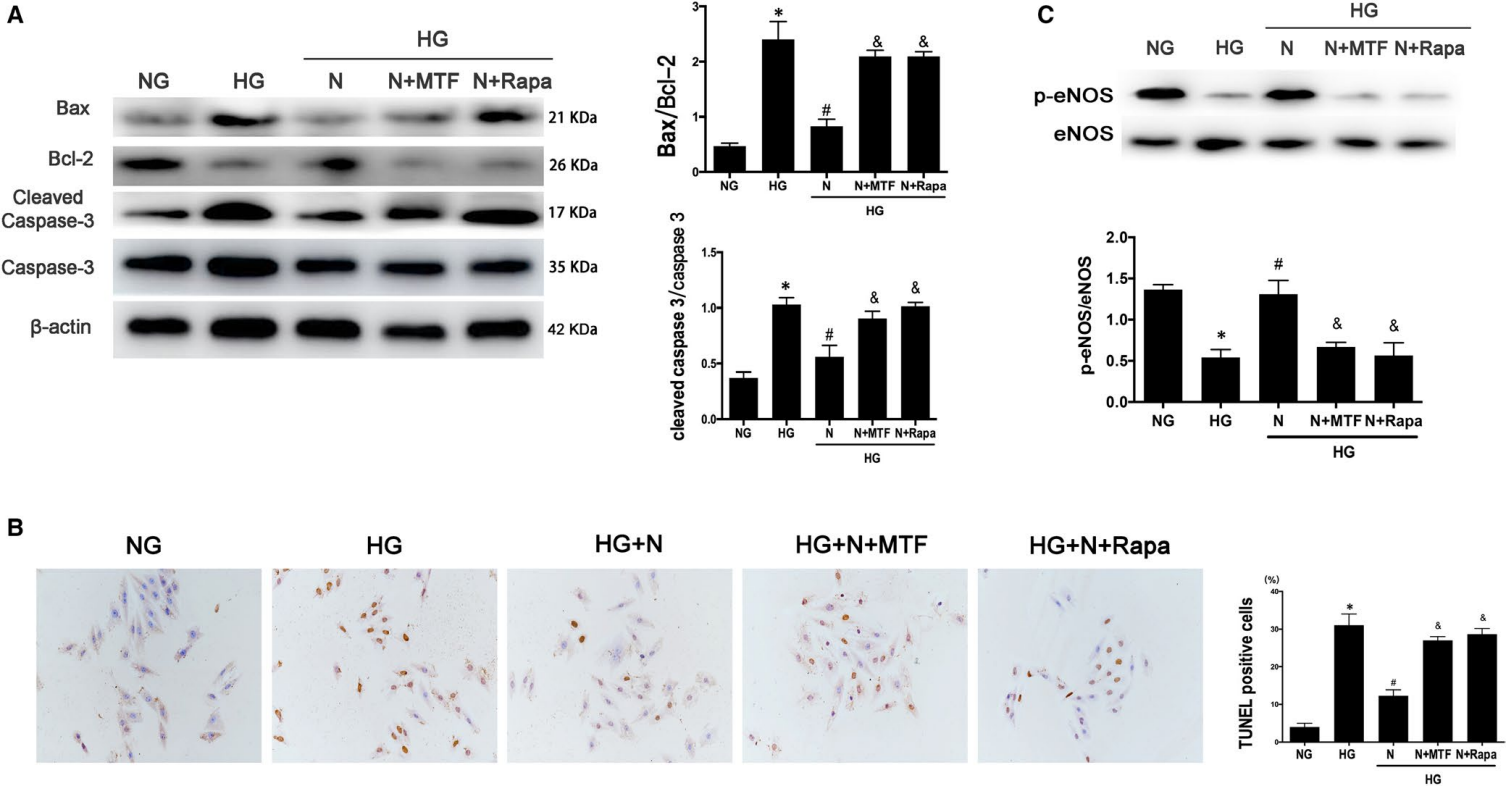
PI3K/AKT pathway inhibition blocked the protection of nicorandil on H9c2 cardiomyocyte treated with high glucose. A: Western blot analysis of Bax/Bcl‐2 and cleaved caspase‐3 in high glucose‐induced H9c2 cardiomyocyte after nicorandil treatment or both nicorandil treatment and PI3K/mTOR inhibitors. B: TUNEL assay of apoptosis rate of high glucose‐induced H9c2 cardiomyocyte after nicorandil treatment or both nicorandil treatment and PI3K/mTOR inhibitors (scale bar: 20 µm). C: Western blot analysis of p‐eNOS in high glucose‐induced H9c2 cardiomyocyte after nicorandil treatment or both nicorandil treatment and PI3K/mTOR inhibitors. N: Nicorandil (100 µmol); MTF: miltefosine (100 µmol); Rapa: rapamycin (100 µmol) NG: normal glucose (5.5 mmol/L); HG: high glucose (25 mmol/L). *P < 0.05 compared with NG; ^#^ *P* < 0.05 compared with HG; &*P* < 0.05 compared with HG + N, Data are means ± SD

The phosphorylation level of the PI3K/AKT pathway was inhibited in the high‐glucose group compared with the normal glucose group. Compared with the untreated HG group, the phosphorylation level of PI3K/AKT pathway protein was increased in the nicorandil treatment group, and the ratio of Bax/Bcl‐2 and cleaved caspase‐3/caspase‐3 was decreased. After blocking this pathway with PI3K inhibitor and mTOR inhibitor, nicorandil treatment was given, but the ratio of Bax/Bcl‐2 and cleaved caspase‐3/caspase‐3 did not decrease significantly compared with the untreated group, indicating that this pathway plays an important role in nicorandil‐induced reduction of apoptosis. The eNOS expression was also measured, the results showed that the ratio of p‐eNOS/eNOS can increase after the nicorandil treatment and the PI3K inhibitor and mTOR inhibitor blocked this function to some degree. The TUNEL‐positive cells is accordance with the expression of apoptosis related proteins (Figure 8).

## 4 DISCUSSION

Diabetes is a serious global health issue and reveals increased incidence and disease related mortality.^20^, ^21^ DCM is an important complication in diabetes mellitus independent of hypertension, coronary disease, valvular heart disease or other heart diseases.^2^, ^3^, ^22^ It is considered that DCM is an important cause to mortality among all the complications of diabetes.^23^

Nicorandil is a K+‐ATP channel opener with a function of nitrate.^12^ It has been proven that nicorandil has numerous protective effects on the cardiovascular system, including reducing the ventricular preload and afterload, improvement of myocardial perfusion, prevention of Ca2+ overload by opening K+‐ATP channels, anti‐inflammatory and anti‐apoptosis effects.^24^, ^25^ Our study found that nicorandil can increase the level of nitric oxide in the serum of diabetic coronary heart disease (CHD) patients significantly (Appendix 1). However, whether nicorandil can directly play a role in the pathological process of diabetic cardiomyopathy has not been reported. In our study, type 2 diabetic models were established successfully and nicorandil was used on rats 8 weeks after high blood glucose. Dosage of 7.5 mg/kg day and 15 mg/kg day were used in our nicorandil‐treated groups. We found that although nicorandil had no significant effect on blood glucose, it significantly improved myocardial remodelling by reducing the ratio of heart weight/bodyweight and decreasing the transverse diameter of myocardial cells in the diabetic rats. These indicate that nicorandil treatment has functions to improve cardiac hypertrophy and myocardial remodelling. Cardiac ultrasound found that there are improvements in systolic and diastolic function in nicorandil‐treated groups compared with untreated group. All these results suggest that nicorandil of the dosage of 7.5 mg/kg day and 15 mg/kg day can protect the heart in diabetic rats, but not through decreasing blood glucose directly.

So we wondered the potential mechanism in the function of nicorandil on DCM. We measured the fibrosis and apoptosis level in the heart of four groups and found that after the treatment of nicorandil, the level of fibrosis and apoptosis in the heart decreased significantly compared with untreated diabetic group, indicating that nicorandil can alleviate the process of fibrosis and apoptosis in the heart of DM. We also measured the NO and ADMA levels in serum and found that nicorandil can promote the level of NO and reduce the ADMA in serum compared with the untreated diabetic rats. Also, the ratio of p‐eNOS/eNOS increased in the nicorandil‐treated group.

All results in vivo suggest that the nicorandil treatment and the decrease of the apoptosis level in DCM are related. Previous studies have found that apoptosis plays an important role in the progression of diabetic cardiomyopathy.^26^, ^27^ Although nicorandil cannot decrease blood glucose directly， it can alleviate the apoptosis in cardiomyocyte resulted from the hyperglycaemia through other pathway. It is known that the production of NO is reduced and eNOS activity is weakened in the environment of hyperglycaemia.^28^ These changes can cause endoplasmic reticulum stress and inflammatory reaction in cardiomyocytes, which promote hypertrophy of cardiomyocyte and apoptosis in the heart and accelerate the process of DCM.^29^ As a NO donor, nicorandil can not only increase NO. We found that the activity of eNOS which can protect cardiomyocytes from apoptosis is also increased after nicorandil treatment. However, the mechanism of nicorandil increases eNOS expression activity in a diabetic environment needs further investigations.

Recent studies have shown that PI3K/Akt pathway can inhibit cell apoptosis and promote cell survival through various pathways.^30^, ^31^ One of these pathways is PI3K/Akt pathway which can promote the phosphorylation of eNOS and mTOR, and eNOS further promote the production of NO.^32^ Previous studies have shown that nicorandil can alleviate cardiomyocyte apoptosis and improve cardiac function through the PI3K/Akt pathway in a rat model of ischaemic reperfusion.^15^, ^33^ In the model of coronary microcirculation obstruction, nicorandil can also alleviate apoptosis in heart.^34^ So we are interested in the precise mechanism of how nicorandil modulates the eNOS‐related signals and further regulates apoptosis. And it is a focus on the role of PI3K/Akt pathway in the process of nicorandil regulates eNOS and the apoptosis in DCM.

In the H9c2 cardiomyocytes which were stimulated with high glucose, the level of TUNEL‐positive cells, the ratio of Bax/Bcl‐2 and cleaved‐caspase‐3/caspase‐3 decreased significantly after treated with nicorandil, 5‐HD ( a kind of nicorandil inhibitor) can inhibit this anti‐apoptotic effect of nicorandil. Also, we found that nicorandil and L‐arginine can synergistically alleviate the eNOS mediated apoptosis with high glucose (Appendix 2). We examined the PI3K/Akt pathway protein levels and found that the phosphorylation level of the PI3K/Akt pathway was reduced after high‐glucose stimulation, which is consistent with previous studies. Interestingly, when H9C2 cardiomyocyte was stimulated with high glucose, the phosphorylation level of this pathway was raised after nicorandil intervention. When we used PI3K/Akt pathway inhibitors on H9C2 cardiomyocyte, we examined the levels of apoptosis‐related proteins and found that the anti‐apoptotic effect of nicorandil disappeared after inhibition of the PI3K/Akt pathway.

In conclusion, it is more clear about the mechanism of how nicorandil protects high‐glucose exposed cardiomyocytes from apoptosis. Nicorandil can produce NO which takes part in various actions to protect myocardial damage in diabetes. Moreover, we have also found that nicorandil can promote eNOS activity in the process of protecting cardiomyocytes. In the environment of high glucose, nicorandil can promote the phosphorylation levels of eNOS, mTOR which can be blocked in HG by activating the PI3K/Akt pathway. These proteins play an important role in inhibiting apoptosis and alleviating DCM. However, it still requires further investigation to explore the specific mechanism of nicorandil in regulating the PI3K/Akt pathway.

## 5 CONCLUSION

In summary, nicorandil plays an important role in the progression of diabetic cardiomyopathy; it can directly relieve cardiac remodelling and improve cardiac function through inhibiting fibrosis and apoptosis in the heart of type 2 diabetic rats. Nicorandil can not only release NO directly but also promote the activity of eNOS and mTOR by increasing the phosphorylation of PI3K/Akt pathway. These mechanisms play an important role in the process of nicorandil alleviating apoptosis in cardiomyocyte caused by hyperglycaemia.

## CONFLICT OF INTEREST

The authors confirm that there are no conflicts of interest.

## AUTHORS' CONTRIBUTIONS

J. Pan and X. Wang were responsible to induce animal model and cell experiment. J. Tian performed immunohistochemistry staining and other staining. D. Liu and M. Zhang analyzed and interpreted the animal model data. Tao Jin and Ming Liu performed the ultrasonic cardiogram examination of heart. F. An was a major contributor in writing the manuscript and western blot experiment. All authors read and approved the final manuscript.

## ETHICS APPROVAL AND CONSENT TO PARTICIPATE

All experimental protocols related to animals were approved by the Shandong University Animal Care Committee.

## CONSENT FOR PUBLICATION

Not applicable.

## DATA AVAILABILITY STATEMENT

All data generated or analysed during this study are included in this published article and its additional information files.

## Figures and Tables

**Figure A1:**
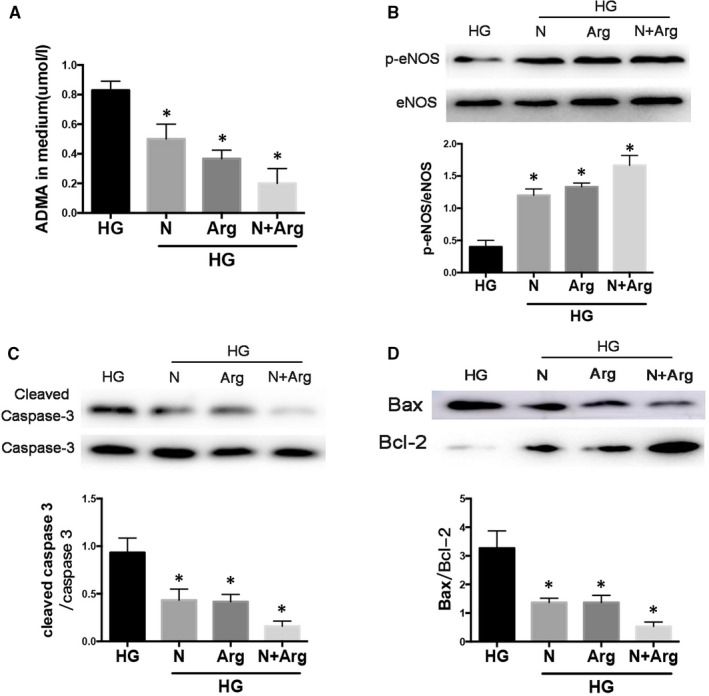
Nicorandil can increase the NO in serum in diabetic coronary heart disease (CHD) patients. A: Basic characteristic of the selected diabetic CHD patients. B: Level of nitric oxide in serum. Nicorandil (5mg tid); *P<0.05 compared with Control.

**Figure A2:**
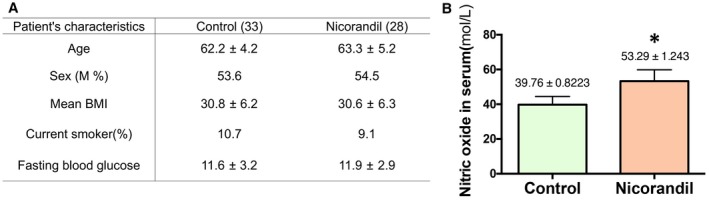
Nicorandil and L‐arginine can synergistically alleviate the eNOS mediated apoptosis in H9C2 cells treated with high glucose. A: ADMA level in medium. B: Western blot analysis of p‐eNOS/eNOS. C: Western blot analysis of caspase‐3. D: Western blot analysis of Bax/bcl‐2. HG: high glucose (33mol/l); N: Nicorandil (100umol); Arg: L‐arginine (75umol). *P<0.05 compared with HG.
